# Measurement properties of the Western Ontario Shoulder Instability Index in Dutch patients with shoulder instability

**DOI:** 10.1186/1471-2474-15-211

**Published:** 2014-06-20

**Authors:** Just A van der Linde, W Jaap Willems, Derk A van Kampen, Loes W A H van Beers, Derek FP van Deurzen, Caroline B Terwee

**Affiliations:** 1 Department of Orthopaedic Surgery and Traumatology, Onze Lieve Vrouwe Gasthuis, Postbus 95500, 1090 HM Amsterdam, the Netherlands; 2 Department of Orthopaedic Surgery and Traumatology, de Lairesse Kliniek, Amsterdam, the Netherlands; 3 Department of Orthopaedic Surgery and Traumatology, Waterland Ziekenhuis, Purmerend, the Netherlands; 4 Department of Epidemiology and Biostatistics and the EMGO Institute for Health and Care Research, VU Medical Center, Amsterdam, the Netherlands

**Keywords:** Validation, Western Ontario Shoulder Instability index, Shoulder, Instability, Measurement properties, Dutch

## Abstract

**Background:**

The Western Ontario Shoulder Instability index (WOSI) is a patient-reported outcome measure for patients with shoulder instability. The purpose of this study was to validate the WOSI in a Dutch population by evaluating its structural validity, internal consistency, measurement error, reliability, and construct validity. Floor and ceiling effects were also addressed.

**Methods:**

Two cohorts were recruited, including a total of 138 patients with shoulder instability. Confirmatory factor analysis was used to assess the structural validity and Cronbach’s α to assess internal consistency. The measurement error was calculated as the smallest detectable change (SDC). Reliability (test–retest) was estimated in a subgroup of 99 patients who completed the re-test after a mean of 13 days (5–30 days). Reliability was calculated with the intraclass correlation coefficient (ICC). Construct validity was evaluated by comparing the WOSI with the Oxford Shoulder Instability Score (OSIS), the Simple Shoulder Test, the Oxford Shoulder Score, the Disability of the Arm, Shoulder, and Hand assessment (DASH), and the Short Form-36 Health Survey. Measurement properties were evaluated for both the total WOSI score and its four domains.

**Results:**

Factor analysis did not confirm the validity of the four domains. Best results were found for a one-factor model. Internal consistency was good, with Cronbach’s α ranging from 0.93 to 0.96. Reliability was excellent (ICC 0.88–0.92 for all subscales). The measurement error (SDC) was 23.0% for the total WOSI and 23% to 28% for the subscales (on a scale of 0–100). Regarding the construct validity, 76% of the results were in accordance with the hypotheses, including a high correlation with the OSIS (0.82) and DASH (0.81) assessments. No floor or ceiling effects were found.

**Conclusion:**

The Dutch version of WOSI showed good reliability and validity in a cohort of patients with shoulder instability, although the factor structure remains unclear.

## Background

With an incidence up to 49/100,000 each year, shoulder instability is commonly seen in orthopaedic clinics [1,2], generally affecting young and active patients [3,4].

Treatment of shoulder instability aims to provide patients with a stable shoulder, enable them to perform overhead activities, and allow them to return to previous (sports) activities. Results of the treatment of shoulder instability are evaluated with both objective and subjective outcome measures. Objective measures include redislocations and range of motion. Subjective measures include questionnaires with regard to shoulder function and are commonly referred to as patient-reported outcomes measures (PROMs).

PROMs are designed to reflect the patient’s subjective function, addressing subdomains such as sports, work, and emotional well-being. They enable the practitioner to detect functional changes in a standardised way. PROMs have become increasingly important in assessing a patient’s health status [5] and can focus on general health, a physical domain or body part (e.g., the shoulder), or a certain condition or disease (e.g., instability) [6].

Several PROMs have been developed over time to evaluate shoulder instability. The Western Ontario Shoulder Instability Index (WOSI) is a well-designed, thoroughly evaluated questionnaire that has proved to be reliable, valid, and sensitive to changes that are of clinical importance to Canadian patients with shoulder instability [7], leading to international acceptance. The WOSI has also been proven useful as an outcome measure in several clinical studies [8-10] and has been translated and validated in Italian, German, Swedish, and Japanese [11-15]. Translation and validation of PROMs allows comparison of national and international study results [7,16-19].

The aim of this study was to translate and validate the WOSI for a Dutch population of patients with shoulder instability. We evaluated its measurement properties according to the *Co*nsensus-based *S*tandards for the Selection of Health *M*easurement *In*struments (COSMIN) guidelines [16].

## Methods

### Translation procedure

The WOSI was independently translated into Dutch by an official translator (Metamorfose Translations, Utrecht, The Netherlands) and three medically educated translators whose native language was Dutch [16,19-21]. When they reached consensus, this version (version 1, or V1) was completed with the help of 20 patients with shoulder instability, who noted whether the questions were applicable to their daily activities. Another 13 patients, who were previously treated for shoulder instability, were asked to complete the Dutch version of the WOSI at home to assess the comprehensibility of the questions. A few linguistic adjustments were made accordingly (V2). These 33 patients were excluded from our final analysis.

This WOSI version was translated back into English by another official translator (Vertaalbureau Oattes, Amsterdam, The Netherlands) and by a native English speaker without a medical background. Both were blinded to the original version and focused on the linguistic aspects. Their versions were compared with the original text. Subsequently, the researchers composed a final version (V3), taking into account all discrepancies.

### Patients and procedures for assessing measurement properties

Two cohorts of patients with shoulder instability were recruited to assess reliability and validity. We planned to include at least 100 patients, which is considered excellent for assessing measurement properties [16,22].

The first cohort included 75 patients with shoulder instability who visited our outpatient clinic between December 2009 and December 2011. The second cohort included 79 patients with shoulder instability who visited the emergency department or the outpatient clinic between December 2012 and May 2013. All patients were recruited at the Onze Lieve Vrouwe Gasthuis, Amsterdam, The Netherlands.

Inclusion criteria were age 16 years or older and any form of glenohumeral instability (anterior, posterior, multidirectional) as diagnosed by one of our doctors. Exclusion criteria were an inability to master the Dutch language and a large glenoid fracture or proximal humeral fracture, such as a displaced fracture of the greater tuberosity. Hill-Sachs and bony Bankart lesions were included. Patients who underwent treatment or follow-up at another clinic were excluded to avoid the inconvenience of a double follow-up.

All patients were assigned a study number and received a web-based questionnaire to be completed at home. All answers were required prior to submission. Patients lacking Internet access received an identical paper version. Missing items were completed by telephone. Patients were asked to complete the questionnaire twice at an interval of 5 to 30 days, which was considered long enough to forget prior answers and short enough to assume an unchanged shoulder condition. Both versions were either web-based or on paper.

The local ethics committee (METC from the Onze Lieve Vrouwe Gasthuis) approved the study and written informed consent was obtained from all patients.

### Patient-reported outcomes measurements

### Western Ontario Shoulder Instability index

The WOSI is a disease-specific PROM developed by Kirkley et al. in [23] according to the methodology described by Kirschner and Guyatt. It was designed to be used as a primary outcome measure in clinical trials that evaluated treatments for patients with shoulder instability [7]. The 21-item questionnaire consists of four domains, referring to physical symptoms, sport/recreation/work function, lifestyle function, and emotional function. Originally responses are given on a 100-mm visual analogue scale, ranging from no complaints (0 mm) to severe complaints (100 mm). We created a web-based version in which patients can choose a score from 0 to 10. Items were summarised in four domain subscores as a total score, ranging from 0 to 2100, where 0 indicated no limitations in shoulder-related quality of life and 2100 indicated extreme limitations. The score could also be expressed as a percentage of normal shoulder function, where a score of 2100 reflected 0% of normal function and a score of 0 reflected 100% [24]. The WOSI was originally validated against the Disabilities of the Arm, Shoulder, and Hand (DASH) assessment and the University of California–Los Angeles (UCLA) shoulder rating scale, with correlations of 0.77 and 0.65, respectively.

### Validation instruments

The following instruments were used to assess the construct of the validity of the WOSI.

#### ***Oxford Shoulder Instability Score***

The Oxford Shoulder Instability Score (OSIS) is a disease-specific PROM developed by Dawson et al. in [21] to assess treatment for shoulder instability. It was originally validated against the Rowe and Constant scores, with correlations of 0.51 and 0.56, respectively. The internal consistency (Cronbach’s α) was 0.92. The reliability was 0.97, calculated with Pearson’s correlation coefficient [21]. The OSIS is currently being translated and validated in Dutch in our institution. Unpublished results show good internal consistency, reliability, and construct validity.

#### ***Simple Shoulder Test***

The Simple Shoulder Test (SST) is a body-part-specific PROM that was developed by Matsen and Lippitt et al. in [25]. It was intended to measure functional limitations of the affected shoulder in patients with common shoulder problems, including rotator cuff tears, degenerative osteoarthritis, and instability [25]. It was validated against the American Shoulder and Elbow Surgeons (ASES) survey with a correlation of 0.81. It has recently been validated in Dutch language, showing high reliability (interclass correlation coefficient (ICC) 0.92) and high internal consistency (Cronbach’s α 0.78) [26,27].

#### ***Oxford Shoulder Score***

The Oxford Shoulder Score (OSS) is a body-part-specific PROM developed in 1996 by Dawson et al. [28,29]. It was developed for patients with general shoulder complaints. The OSS was originally validated against the Constant Shoulder Score and Short Form-36 Health Survey (SF-36) subscales, with correlations of -0.74 and -0.66, respectively (the highest correlation was with the SF-36 Pain subscale). It was later validated in Danish [30], Korean [31], Turkish [32], Italian [33], German [34], and Dutch. It had high reliability (ICC 0.98) and high internal consistency (Cronbach’s α 0.92) [35].

#### ***Disability of the Arm, Shoulder, and Hand assessment***

The Disability of the Arm, Shoulder, and Hand (DASH) assessment is a body-part-specific PROM. It was developed in 1996 by the American Association of Orthopaedic Surgeons to measure physical functions and symptoms in patients with musculoskeletal disorders caused by any condition in any joint in the upper extremity. The DASH was shown to be reliable, valid, and responsive for patients with shoulder disabilities [36]. It was validated by Beaton et al. in [37,38]. The DASH was validated in English against the Shoulder Pain and Disability Index, and correlations with the pain and function subscales were 0.82 and 0.88, respectively. It was also validated in Dutch for patients with disorders of the upper limb. It had high internal consistency (Cronbach’s α 0.95) and reliability (Pearson’s correlation coefficient 0.98) [39].

#### ***Short Form 36 Health Survey, version 1***

The Short Form 36 Health Survey (SF-36) is the most widely used PROM for assessing general health [40]. It has eight domains: Physical function, Social function, role limitations caused by physical problems (Role physical), role limitations caused by emotional problems (Role emotional), General mental health, Vitality, Bodily pain, and Perception of general health [41]. The SF-36 was translated and validated in a Dutch general population [14]. Previous studies have also validated the SF-36 specifically for shoulder complaints [42,43].

### Assessment of measurement properties

### Structural validity and internal consistency

Items of PROMs that are being summarised into one score (either a subscale or total score) should measure the same construct. Structural validity is defined as the degree to which the scores of an instrument are an adequate reflection of the dimensionality (i.e., expected number of subscales) of the construct to be measured [17]. Thus, in case of the WOSI, do questions within the subscales measure the same construct (e.g., physical symptoms, sport/recreation/work function, lifestyle function, emotional function)? Likewise, do questions from different subscales measure different constructs?

Structural validity was assessed by confirmatory factor analyses (CFA) using baseline measurements. We expected four factors—one for each of the WOSI domains. Factor loadings represent the correlation between the items in the questionnaire and the factors (the underlying dimensions). We examined factor loadings and model fit with CFA for categorical items, performed in Mplus (modelling program) using the method of weighted least squares with mean and variance adjustment.

Factor loadings are generally considered to be meaningful when they exceed 0.30 or 0.40 [44]. We considered factor loadings of at least 0.50 appropriate. The Comparative Fit Index (CFI), Tucker-Lewis Index (TLI), and the Root Mean Square Error of Approximation (RMSEA) were used as measures for model fit. A CFI and TLI of >0.95 and a RMSEA of <0.05 were considered as adequate fit. For moderate fit, values >0.90 and <0.08 were used [45]. Because the model did not fit well (see Results), additional exploratory factor analyses were performed with SPSS software (SPSS Inc., Chicago, IL, USA), using the Varimax rotation.

Internal consistency is defined by COSMIN as the degree of interrelatedness among the items [17]. Items may ask similar questions in slightly different ways for reliably capturing the respondent’s opinion or level of function [29]. The internal consistency of the WOSI was assessed by calculating Cronbach’s α for each subscale. Cronbach’s α is preferably ≥0.70 [46].

### Measurement error and reliability

The measurement error is the systematic and random error of a patient’s score that is not attributed to true changes in the patient’s condition [17]. When a patient’s score changes within the range of the measurement error, it is unclear whether the change is an effect of the therapy or should be attributed to a measurement error.

Measurement error can be expressed as the standard deviation of repeated measurements in a single patient, which is referred to as the standard error of measurement (SEM). The SEM was calculated from the square root of the variance between the measurements and the error variance of the ICC. Subsequently, the SEM can be transformed into the smallest detectable change (SDC = 1.96*√2*SEM), which can be used to interpret change scores in individual patients over time. It represents the minimum change a patient must show to ensure that the observed change is real and not a measurement error [47].

Reliability is defined as the proportion of the total variance in the measurements that is due to true differences between patients [17]. Reliability is calculated using the ICC with a two-way mixed-effects model for absolute agreement. The ICC ranges from 0 (poor reliability) to 1 (patients with unchanged health status whose answers would be the same on two occasions). Scores ≥0.70 are considered adequate [46].

### Construct validity

Construct validity refers to the degree to which scores are consistent with hypotheses regarding relations with other instruments measuring similar constructs. In this study, the condition-specific WOSI was compared with the OSIS, measuring a similar disease-specific construct (shoulder instability); the SST, OSS, and DASH, assessing a similar body-specific domain (shoulder); and several subscales of the original version of SF-36, measuring general health status. The hypotheses were based on clinical experience, knowledge of several PROMs, and consensus among the study investigators. Our hypotheses are presented in Table 1.

**Table 1:** Predetermined hypotheses for testing the validity of the Dutch version of WOSI: expected and observed correlations

	**Expected correlations**	**Observed correlations**
1. WOSI and OSIS	≥ 0.7	0.82
2. WOSI and SST	≥ -0.6	- 0.66
3. WOSI and OSS	≥ 0.6	0.79
4. WOSI and DASH	≥ 0.6	0.81
5. Correlation between WOSI and OSIS, both measuring a disease-specific construct should be at least 0.1 higher compared with all other correlations.		
6. Correlation between similar WOSI and SF-36 domains should be higher compared with dissimilar domains.		
7. Correlation between similar WOSI and SF-36 domains should be ≥ 0.4.		

Correlations were calculated using the total WOSI score.

Expected correlation between the WOSI and the OSIS was ≥0.70. Between the WOSI and the SST, OSS, and DASH assessment it was ≥0.60. The highest correlation was expected between two PROMs assessing the same disease-specific construct (WOSI and OSIS, both measuring limitations caused by shoulder instability).

Each WOSI domain was expected to have the highest correlation with its comparable SF-36 domain: WOSI Physical symptoms and SF-36 Bodily pain; WOSI Sport/recreation/work and SF-36 Role functioning; WOSI Lifestyle and SF-36 Social functioning; WOSI Emotional function and SF-36 Mental health. These four correlations were also expected to be at least 0.40.

In total, 79 correlations (or comparisons between correlations) were evaluated. Construct validity was considered good when at least 75% of the results were in accordance with our hypotheses [48].

### Floor and ceiling effects

Floor and ceiling effects occur when more than 15% of patients achieve the lowest or highest possible score, respectively [49]. When patients already have the highest or lowest possible score before intervention, it is impossible to measure further improvement or deterioration.

When we take the SDC into account we should consider floor and ceiling effects more broadly. If a score is close to one of the extremes, and the distance between the initial score and the extreme is smaller than the SDC, a change beyond the measurement error cannot be measured. For this reason, we also assessed how many scores were observed within the SDC range from both extremes.

### Statistical analyses

Statistical analyses were performed using SPSS software version 18.0.0 and MPlus.

## Results

### Translation process

Forward translation of the WOSI into Dutch (V1) did not impose any problems. No difficulties occurred with the patients completing the questionnaire under supervision or at home. Their answers were not used in the subsequent validation process. Translating the WOSI backward also did not impose any problems.

### Patients

**Figure 1:**
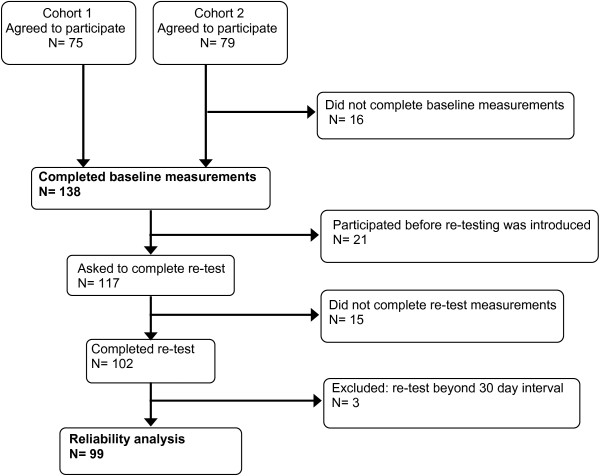
Flowchart showing selection of patients who participated in the study.

A total of 154 patients with shoulder instability were recruited, among whom 138 patients (90%) completed the WOSI. Because retesting was initiated after the first 21 patients had been included, 117 were asked to complete the WOSI twice. Fifteen patients were either not able or not willing to participate in the retest. Of the remaining 102 patients, three were excluded because they exceeded the 30-day interval. In total, 99 (64%) completed a retest. A flowchart is presented in Figure 1. The mean time between completion of the first and second questionnaires was 13 days (5–30 days). Sixteen patients completed their first questionnaire on paper followed by a web-based retest; four patients completed both questionnaires on paper.

Table 2 shows the patients’ demographic data and the mean scores of all PROMs at baseline and at retesting. The mean age at baseline was 32 years. Men were affected more frequently than women. Both sides of the shoulder were equally affected. All patients had suffered anterior dislocations. As measured with the OSIS, OSS, SST, and DASH evaluations, there was no significant change in shoulder function at baseline and retesting.

**Table 2:** Demographic data and data from the OSIS, SST, OSS, and DASH at baseline and retesting

		**Baseline assessment**	**Reliability cohort**
		138	99
Mean age yrs (SD)		32 (12)	32 (14)
Gender (male vs female)		98 (71%) vs 40 (29%)	66 (67%) vs 33 (33%)
Dislocated shoulder			
Right		72 (53%)	54 (55%)
Left		59 (43%)	40 (40%)
Both		6 (4%)	5 (5%)
Dominant side dislocated		72 (53%)	53 (54%)
Date first dislocation			
<1 month		8 (6%)	8 (8%)
1 - 6 months		21 (15%)	17 (17%)
>6 months – 2 years		40 (29%)	25 (25%)
>2 years		67 (49%)	49 (50%)
OSIS	(0 – 48)*	27.3 (9.1)	27.6 (9.7) ¶
SST	(0 – 12)*	8.8 (3.1)	8.8 (3.2) ¶
OSS	(48 – 0)*	23.7 (7.8)	22.8 (8.3) ¶
DASH	(100 – 0)*	22.2 (16.7)	22.7 (18.3) ¶

*ranges reflect most impaired to least impaired function.

¶No significant change in shoulder function (OSIS, SST, OSS, DASH) was observed at retest compared with baseline.

### Structural validity and internal consistency

The expected four-factor model did not fit well: CFI 0.869, TLI 0.850, RMSEA 0.104. Subsequently, three-factor, two-factor, and one-factor models were tested by exploratory factor analyses (Table 3). The best interpretable results were found with only one factor, although confirmative testing of this one-factor model in CFA showed worse fit (CFI 0.800, TLI 0.778, RMSEA 0.127) than the original four-factor model.

**Table 3:** Exploratory factor analysis

	**3-factor**			**2-factor**		**1-factor**
**Question**	**Factor 1**	**Factor 2**	**Factor 3**	**Factor 1**	**Factor 2**	**Factor 1**
1	,302	,402	**,662**	**,743**	,357	**,779**
2	,145	,375	**,761**	**,830**	,192	**,725**
3	,396	**,604**	,417	**,625**	**,531**	**,818**
4	,339	**,546**	**,523**	**,693**	,449	**,809**
5	,125	**,653**	,296	**,571**	,300	**,617**
6	,152	**,560**	,470	**,671**	,281	**,675**
7	,028	,375	**,706**	**,793**	,086	**,624**
8	,407	**,665**	,230	,494	**,577**	**,757**
9	,323	**,704**	,297	**,579**	**,504**	**,766**
10	,374	**,648**	,332	**,576**	**,531**	**,783**
11	**,659**	,451	,299	,423	**,740**	**,822**
12	**,608**	,469	,396	**,520**	**,690**	**,855**
13	**,694**	**,533**	,075	,269	**,819**	**,767**
14	,375	,147	**,738**	**,674**	,338	**,717**
15	**,582**	,378	,185	,295	**,654**	**,670**
16	**,535**	,020	**,578**	,458	,463	**,652**
17	**,624**	,402	,156	,279	**,704**	**,693**
18	,204	,277	**,732**	**,749**	,219	**,686**
19	**,753**	,327	,113	,192	**,805**	**,703**
20	**,792**	,051	,173	,102	**,749**	**,600**
21	**,738**	,155	,364	,323	**,715**	**,733**
Scores						
≧ 0.50	9	8	7	12	12	21

Factor loadings ≥0.50 are appropriate (bold). Best results are with the 1-factor model in which all questions score at least 0.50.

Internal consistency was analysed using baseline measurements for all 138 patients. For the WOSI domains, Cronbach’s α was 0.93 for emotional function, 0.94 for physical symptoms and lifestyle function, and 0.95 for sports/ recreation/work. For the WOSI total, Cronbach’s α was 0.96.

### Measurement error

For the total WOSI score, the SEM was 8.3%, resulting in an SDC of 23.0%. This indicates that a patient has to change 23.0 points on a scale from 0 to 100 to detect an actual change in shoulder function (that cannot be attributed to measurement error). For the domains, the SEM varied from 8.3% to 10.1%, resulting in an SDC ranging from 23.1% to 28.1%. Scores are presented in Tables 4 and 5.

**Table 4:** Test–retest reliability (ICC) and the standard error of measurement (SEM) for the WOSI

**N = 99**	**Mean (SD)**			**SEM**	**ICC (95% CI)**
	**Baseline**	**Re-test**	**Change**		
WOSI					
Total	971 (482)	959 (509)	-12.1 (199.5)	174	0.92 (0.88-0.95)
Total as %	46.0 (22.3)	45.7(24,2)	- 0.6 (9.5)	8.3	0.92 (0.88-0.95)
Domain as %					
Physical	60.2 (24.0)	60.6 (24.7)	0.4 (11.2)	8.3	0.90 (0.85 – 0.93)
sp/rec/wrk	47.7 (27.8)	49.6 (28.5)	1.9 (12.7)	9.4	0.90 (0.85 – 0.93)
Lifestyle	56.5 (24.3)	55.8 (25.4)	- 0.7 (12.3)	8.8	0.88 (0.83 - 0.92)
Emotion	36.8 (27.5)	37,8 (30.2)	1.0 (14.4)	10.1	0.88 (0.82 - 0,92)

Scores are presented for the total WOSI score (range 0–2100), the total WOSI score expressed as a percentage (range 1–100), and all WOSI subdomains.

**Table 5:** Floor and ceiling effects and the smallest detectable change in the WOSI

**N = 138**	**Absolute floor**	**Absolute ceiling**	**SDC**	**SDC- range from**		**% of patients scoring within SDC -range**	
**WOSI**				**Floor**	**Ceiling**	**Floor**	**Ceiling**
total	No	No	23.0	0-23	77-100	17%	5%
Domain							
physical	No	No	23.1	0-23.1	76.9-100	3%	26%
sp/rec/wrk	No	No	26.0	0-26	74.0-100	23%	20%
lifestyle	No	No	24.4	0-24.4	74.6-100	9%	25%
emotion	No	No	28.1	0-28.1	71.9-100	41%	10%

From left to right are presented; absolute floor and ceiling scores. The smallest detectable change (SDC) with its ranges, and the percentage of scores that fell within the SDC-range for both extremes. Scores are presented for the total WOSI score and all WOSI subdomains.

### Reliability

The WOSI test and retest scores are shown in Table 4. The mean WOSI total score at baseline was 971 (46.0%). The mean total WOSI retest score was 959 (45.7%). For the four domains, scores expressed as a percentage ranged from 60.2% to 82.6% at baseline and from 37.8% to 60.6% at retest.

Regarding the WOSI total, the ICC (95% CI) was 0.92 (0.88–0.95), indicating excellent reliability. For the WOSI domains, the ICC ranged from 0.88 (0.82–0.92) to 0.90 (0.85–0.93).

### Construct validity

Correlations are summarised in Table 1. The correlation between the instability-specific WOSI and OSIS was 0.82 (≥0.70 expected). The correlations between the WOSI and the shoulder-specific SST, OSS, and DASH were -0.66, 0.79, and 0.81, respectively (≥0.60 expected).

The correlations between both the total WOSI score and WOSI domains and the SF-36 domains are presented in Table 6. The correlation between the WOSI and OSIS was at least 0.10 higher than all other correlations, except the correlation between the total WOSI score and SF-36 Bodily pain (0.76) and between the WOSI Physical functioning domain and SF-36 Bodily pain (both 0.76).

**Table 6:** Observed correlations between the WOSI domains and SF-36 domains

	**WOSI**	**Physical**	**sp/rec/work**	**Lifestyle**	**Emotions**
	**Total**				
SF-36					
PF	-0.69	0.63	0.71	0.66	0.46
RF	-0.60	0.56	**0.60**	0.54	0.40
RE	-0.48	0.44	0.42	0.46	0.37
MH	-0.28	0.23	0.23	0.30	**0.27**
V	-0.39	0.35	0.31	0.45	0.31
SF	-0.51	0.46	0.51	**0.46**	0.39
BP	-0.76	**0.76**	0.71	0.67	0.46
GH	-0.36	0.35	0.28	0.35	0.27

Expected correlations ≥ 0.4 between similar domains are expressed in bold.

*PF* = Physical Functioning, *SF* = Social Functioning, *GH* = General Health, *V* = Vitality, *MH* = Mental Health, *RE* = Role Emotional, *RF* = Role Functional, *BP* = Bodily Pain.

Correlations between similar WOSI and SF-36 domains were highest, as expected, except for WOSI Emotional function (0.27). Three of four correlations between similar WOSI and SF-36 domains were at least 0.40.

In total, 76% of the results were in accordance with the hypotheses.

### Floor and ceiling effects

Floor and ceiling effects are presented in Table 5. No floor or ceiling effects were found. When considering the SDC, however, more than 15% of the scores in two subdomains were within the SDC from the lowest possible score (23% and 41%), and more than 15% of the scores in three subdomains were within the SDC from the highest possible score (20%, 25%, 26%).

## Discussion

International adoption and validation of measurement tools helps us to exchange results globally in a standardised way, thereby enabling international evaluation to optimise treatment strategies. Regarding shoulder instability, the WOSI is the most thoroughly studied PROM to evaluate shoulder functioning in patients with shoulder instability. It has officially been validated in five other languages since its development in English. Measurement properties of the original WOSI and subsequent validation studies are summarised in Table 7.

**Table 7:** Measurement properties of the WOSI as presented in the original article and subsequent validation studies

**Study**	**N**	**Internal consistency**	**ICC (interval)**	**Construct validity**		**SEM and SDC**	**Floor/ ceiling**	**MIC**	**SRM & ES**
Kirkley et al. [7]	Total: 300	N = 33	N = 51	N = 47 (baseline)		NA	NA	NA	SRM 0.931
	Not specified	item reduction	0,494 (2wks)	DASH	0.768				
			0.911 (3mnts)	UCLA	0.649				
				Constant	0.590				
				Rowe	0.609				
				ASES	0.553				
				SF12 physical	0.656				
				SF-12 mental	0.115				
				ROM	0.394				
Salomonsson et al. [11]	Total: 99	N = 22	N = 32	N = 22		NA	Not found	NA	N = 22
	22 surgery	α = 0.89	0.94 (2 mnts)	VAS function	0.80				SRM 1.40
	32 partly surgery			EQ-5D	0.44				ES 1.67
	45 healthy			N = 32 Rowe	0.59				
Hatta et al. [13]	Total: 85	N = 85	N = 59	N = 85		NA	NA	NA	NA
	Not specified	α = 0.84	0.91 (2wks)	Quick DASH	0.63				
				Rowe	0.42				
				SF-36					
				Physical funct	0.36				
				Social funct	0.10				
				General health	0.27				
				Mental health	0.26				
				Vitality	0.28				
				Role emotional	0.16				
				Role functional	0.26				
				Bodily pain	0.34				
Hofstaetter et al. [12]	Total: 86	N = 24	N = 25	Rowe	0.627	NA	High ceiling in healthy shoulders	NA	NA
	24 surgery	α = 0.92	0.92 (24–72 hr)	UCLA	0.609				
	25 partly surgery			Constanst	0.590				
	37 healthy			SF-36					
				Physical funct	0.44				
				Social funct	0.32				
				General health	0.34				
				Mental health	0.38				
				Vitality	0.33				
				Role emotional	0.32				
				Role functional	0.39				
				Bodily pain	0.56				
Drerup et al. [14]	Total: 30	N = 29	N = 29	ASES	0,58	NA	Not found	NA	NA
	Not specified	α = 0.89	0.87						
Cacchio et al. [15]	Total: 64	N = 64	N = 64	DASH	0.79	SEM 71	Not found	40	N = 39
	Not specified	α = 0.93	0.95 (3 days)	SF-36	0.11	SDC 196		0	SRM 1.94
			N = 20						ES 1.47
			0.92 (14 wks)						

Measurement properties include the internal consistency (Cronbach’s α), intraclass correlation coefficient (ICC), construct validity (Pearson’s correlation coefficient), standard error of measurement (SEM), smallest detectable change (SDC), floor- and ceiling effects, minimum important change (MIC), and sensitivity to change [standardised response mean (SRM) and effect size (ES)]. NA means not assessed.

Translating the WOSI into Dutch did not incur difficulties and resulted in a well-translated and comprehensive Dutch version.

Regarding the structural validity, we were unable to confirm the validity of the four domains of the WOSI. An exploratory factor analysis suggested a one-factor model, but this model fit even worse. The factor structure and the value of the four domains of the WOSI therefore remain unclear. Apparently, there is no clear distinction between the questions about symptoms, physical functioning, and emotional aspects. Also, when reading the questions, there is a lack of face validity of the four dimensions. For example, questions about fear of falling or sleeping are included in the lifestyle subscale, which may actually measure emotional aspects and symptoms, respectively. Also one may wonder whether a question about ‘feel the need to protect your arm during activities’ refers to functioning or emotional aspects. The subscales should therefore be used with caution.

A high Cronbach’s α of 0.96 for the total WOSI score and 0.93–0.95 for the subscales were found, which exceeded those in previous validation studies (ranging from 0.84 to 0.93). Compared with other Dutch-validated PROMs, Cronbach’s α of the WOSI was higher than that of the SST (0.78), OSS (0.92), or DASH (0.95) [26,35,39]. However, Cronbach’s α of the WOSI total score was highly affected by the large number of items.

This study is the second one to report on measurement errors of the WOSI. Cacchio et al. [15] reported an SEM of 71 and an SDC of 196 in 64 patients. We found much higher SEM and SDC values (174 and 483, respectively), indicating that a patient has to improve at least 23% of the total score (483/2100 possible points) to ensure an improvement beyond measurement error. It should be noted that the SDC refers to the measurement error in one changed score in one individual patient. When measuring change in a group of patients (as in a study), the measurement error of the mean change score is much lower (in fact, SDC/√n).

With an ICC of 0.92 for the total WOSI score and 0.88–0.90 for the subscales, the reliability of the Dutch version is considered very good. Including 99 patients in our test–retest analysis, our population was larger than populations described in previous validation studies (25–64 patients).

Our study is most similar to those performed by Kirkley et al. and Hatta et al. [7,13] regarding both the length of the test–retest interval (both 2 weeks) and the size of the patient population (51 and 59 patients, respectively). These studies reported ICCs of 0.94 and 0.91, respectively, for the WOSI total score.

Studies performed by Salomonsson et al., Hofstaetter et al., and Cacciho et al. [11,12,15] all had smaller patient populations (32, 25, and 30, respectively) and differed in their treatment-free test–retest interval. Hoffsaetter et al. and Cacchio et al. used a test–retest interval of 24–72 h and 3 days or 14 weeks, respectively. Salomonsson et al. used an interval of 2 and 3 months, respectively. These studies nevertheless present comparable ICCs for the total WOSI score, varying from 0.91 to 0.95. Only Drerup et al. [14] reported a lower ICC (0.87), without defining either its test–retest interval or patient population.

To assess the construct validity, Kirkley et al. calculated correlations with the DASH, the UCLA shoulder rating scale, the Constant score, the Rowe rating scale, ASES, and SF-12. The original Rowe and Constant scores are not PROMs but observer-based measurement instruments, and the Constant score is considered not applicable to shoulder instability [50,51]. We used only PROMs for the Dutch validation. Because the SST and OSS are validated in Dutch, and because preliminary results of the Dutch OSIS validation are good, we decided to use these instruments instead of the UCLA shoulder rating scale and ASES. It should be noted, however, that the WOSI is validated against the OSIS, and the OSIS is validated against the WOSI. Unfortunately, there is no gold standard or other validated PROM for shoulder instability that could be used to assess construct validity. Therefore, we chose this method but also included other instruments. The high correlation between WOSI and OSIS (0.82) means that the two questionnaires are measuring the same construct, but it does not guarantee that both instruments are valid.

With 76% of our predetermined hypothesis being confirmed, construct validity was considered good.

Despite the fact that few questions of the DASH assessment and WOSI overlap, a high correlation was observed (0.81). Both the original article [7] and studies using WOSI translations in Japanese and Italian also found a higher correlation with the DASH and Quick DASH than with other outcome measures (0.77, 0.63, and 0.79, respectively).

Regarding the total WOSI score, no floor or ceiling effects were found, as also described by McHorney [49]. When the SDC (23.0%) is taken into account, however, a total of 23 scores (17%) were within the SDC from the lowest possible score. No real deterioration beyond measurement error could be detected in these patients.

A strong aspect of this study is our large population of patients with shoulder instability and without missing values regarding the PROM questions. Although needed to perform this study, a weak aspect might be the total number of questions posed to our patients. Completing six questionnaires at once requires considerable time and concentration, during which patients might lose their focus. Another weak point is the fact that we used a preliminary version of the Dutch OSIS to validate the WOSI. Official translation and validation is a subject of future, yet unpublished studies in our institution.

Future studies should focus on determining the responsiveness and the minimum important changes (MIC) needed in the WOSI. This information can be used to determine whether observed changes are important to patients and to determine the number of patients who achieve a change greater than the MIC (e.g., responders in an intervention study). The numbers of responders can then be compared between groups in clinical trials [52].

## Conclusion

The Dutch version of the WOSI showed good reliability and construct validity in a cohort of patients with shoulder instability, but the factor structure remains unclear.

## Competing interests

The authors declare that they have no competing interests.

## Authors’ contributions

JL is the principal investigator; he included patients, analysed data and is the author of the first and consecutive drafts of the manuscript. DK created the study design, he included patients in the first cohort, assisted with the analysis and obtained ethical approval. LB created the study design and assisted with data analysis. DD is responsible for the inclusion of patients in the second cohort, participated significantly to the first and consecutive draft of the manuscript. CT is responsible for the study design and participated significantly in the analysis and interpretation of the results. JW is responsible for the study design and is responsible for the inclusion of patients in the second cohort. All authors read and approved the final manuscript, to which they all attributed significantly.

## Pre-publication history

The pre-publication history for this paper can be accessed here:

http://www.biomedcentral.com/1471-2474/15/211/prepub
